# PROGRAM OF ALL INCLUSIVE CARE FOR THE ELDERLY ORGANIZATIONS FLIP THE SCRIPT IN RESPONSE TO COVID-19

**DOI:** 10.1093/geroni/igad104.3537

**Published:** 2023-12-21

**Authors:** Morgan Perry, Stephen McCall, Joseph Dorris, Michael Nardone, Ani Turner, Samuel Obbin, Christine Stanik

**Affiliations:** University of Michigan, Ann Arbor, Michigan, United States; Altarum, Ann Arbor, Michigan, United States; Altarum, Ann Arbor, Michigan, United States; The Nardone Group LLC, Philadelphia, Pennsylvania, United States; Altarum, Ann Arbor, Michigan, United States; Altarum, Ann Arbor, Michigan, United States; Arbor Research Collaborative, Ann Arbor, Michigan, United States

## Abstract

The Program of All-Inclusive Care for the Elderly (PACE) is a unique care delivery model providing comprehensive, community-based, person-centered services to older adults. The COVID-19 pandemic posed unprecedented challenges for PACE organizations to continue providing services while mitigating infection risk, and they were agile and creative in their response. This study leveraged two national surveys and six interviews with PACE directors to examine how PACE organizations met these challenges. The response rate was 71.2% for the first survey and 67.8% for the second. Results revealed a rapid transition of the PACE model from primarily center-based to home-based care. To aid this transition, PACE organizations redeployed staff, repurposed day centers, and leveraged technology, among other strategies. Most PACE organizations plan to continue delivering more services in the home, consistent with participant preferences. The findings from this study indicate that PACE organizations continued to keep participants at the forefront as they transitioned to a home-based model. These findings highlight PACE organizations’ ability to empower nursing-home-eligible older adults to remain living in their preferred residential setting and could inform lasting changes in PACE service delivery.

